# Risk Prediction in Patients With Heart Failure With Preserved Ejection Fraction Using Gene Expression Data and Machine Learning

**DOI:** 10.3389/fgene.2021.652315

**Published:** 2021-03-22

**Authors:** Liye Zhou, Zhifei Guo, Bijue Wang, Yongqing Wu, Zhi Li, Hongmei Yao, Ruiling Fang, Haitao Yang, Hongyan Cao, Yuehua Cui

**Affiliations:** ^1^Division of Health Management, School of Management, Shanxi Medical University, Taiyuan, China; ^2^Division of Health Statistics, School of Public Health, Shanxi Medical University, Taiyuan, China; ^3^Department of Hematology, Taiyuan Central Hospital of Shanxi Medical University, Taiyuan, China; ^4^Department of Cardiology, First Hospital of Shanxi Medical University, Taiyuan, China; ^5^Division of Health Statistics, School of Public Health, Hebei Medical University, Shijiazhuang, China; ^6^Key Laboratory of Major Disease Risk Assessment, Shanxi Medical University, Taiyuan, China; ^7^Department of Statistics and Probability, Michigan State University, East Lansing, MI, United States

**Keywords:** risk prediction, kernel partial least squares, genetic algorithm, heart failure with preserved ejection fraction, machine learning

## Abstract

Heart failure with preserved ejection fraction (HFpEF) has become a major health issue because of its high mortality, high heterogeneity, and poor prognosis. Using genomic data to classify patients into different risk groups is a promising method to facilitate the identification of high-risk groups for further precision treatment. Here, we applied six machine learning models, namely kernel partial least squares with the genetic algorithm (GA-KPLS), the least absolute shrinkage and selection operator (LASSO), random forest, ridge regression, support vector machine, and the conventional logistic regression model, to predict HFpEF risk and to identify subgroups at high risk of death based on gene expression data. The model performance was evaluated using various criteria. Our analysis was focused on 149 HFpEF patients from the Framingham Heart Study cohort who were classified into good-outcome and poor-outcome groups based on their 3-year survival outcome. The results showed that the GA-KPLS model exhibited the best performance in predicting patient risk. We further identified 116 differentially expressed genes (DEGs) between the two groups, thus providing novel therapeutic targets for HFpEF. Additionally, the DEGs were enriched in Gene Ontology terms and Kyoto Encyclopedia of Genes and Genomes pathways related to HFpEF. The GA-KPLS-based HFpEF model is a powerful method for risk stratification of 3-year mortality in HFpEF patients.

## Introduction

Heart failure (HF) is the leading cause of death and disability worldwide among older adults (Manolis et al., 2019). Over 50% of patients with HF exhibit heart failure with preserved ejection fraction (HFpEF; Komajda et al., 2011; Rich et al., 2018), and the prevalence of HFpEF is increasing relative to heart failure with reduced ejection fraction (HFrEF) at an alarming rate of 1% per year (Monika et al., 2018). HFpEF is a heterogeneous syndrome that contributes to abnormal cardiac structure or function, seriously endangering human health (Antlanger et al., 2017; Garg et al., 2017). HFpEF patients have a poor prognosis, and the 5-year mortality rate of HFpEF is as high as 50% (Shah et al., 2017). While the mortality rate of HFrEF has significantly decreased over the past few years because of specific HFrEF treatments (Loh et al., 2013), no effective treatment has been identified for HFpEF patients (Shah et al., 2014). Arguably, with an aging population worldwide, the emerging epidemic of HFpEF requires urgent attention to determine methods for faster disease risk assessment and to predict clinical outcomes to guide therapy, monitoring, and patient management.

While numerous risk assessment models have been developed in cohorts with HFrEF or a mixture of HFrEF and HFpEF, risk prediction in HFpEF patients has been less studied (Thorvaldsen et al., 2017; Angraal et al., 2020). This may be associated with the poor prognostic factors used to predict HFpEF patients (Kanda et al., 2018). The existing risk assessment models for HFpEF are predominantly based on clinical phenotype data, such as baseline demographic and clinical data and electrocardiographic, echocardiographic, and laboratory testing data (Komajda et al., 2011; Thorvaldsen et al., 2017; Rich et al., 2018; Angraal et al., 2020). Unfortunately, these models constructed using clinical phenotypic data have low sensitivity or specificity, and patients are likely to be misdiagnosed. No model has gained widespread acceptance to date. The estimate of an HFpEF patient’s prognosis in daily practice is still mainly based on the experience of clinicians (Ferrero et al., 2015; Thorvaldsen et al., 2017; Manolis et al., 2019). A great need exists to develop an effective risk model for HFpEF to aid in the design of future clinical trials.

With advances in sequencing and computer technology, high throughput expression data can be extracted without limits. Genomic measures of gene expression offer rich information about the underlying disease mechanism and have provided new possibilities of using these molecular data to understand the disease gene function and further predict disease outcomes (Haring and Wallaschofski, 2012). Based on the expression data, great efforts have been devoted to disease classification, clinical outcome prediction, and the identification of genes with potential therapeutic molecular signatures (Penney et al., 2011; Khan et al., 2012; Vargas and Lima, 2013; Wang et al., 2019). HFpEF is a complicated clinical syndrome with high molecular heterogeneity and diverse manifestations (Shah et al., 2015) and is further complicated with a potentially nonlinear relationship between genes and the clinical outcome. Thus, conventional generalized linear models (e.g., logistic regression) are poor choices for risk prediction. Advanced statistical techniques and machine learning methods show great potential in improving the classification performance over conventional statistical tools through the nonlinear effects of variables to achieve accurate prediction (Angraal et al., 2020) and should be studied for HFpEF prediction.

The purpose of this work is to evaluate six different risk stratification models and to predict the survival risk of HFpEF patients based on gene expression profiles using data from a high-quality epidemiologic study, the Framingham Heart Study (FHS). We applied five advanced machine learning methods [i.e., kernel partial least squares based on the genetic algorithm (GA-KPLS), random forest (RF), the least absolute shrinkage and selection operator (LASSO), ridge regression (RR), support vector machine (SVM), and a conventional logistic regression model (Logit)] to build an optimal risk stratification model. Identification of patients with a high risk of HFpEF will be helpful for targeted interventions and clinical trials to further improve the survival of HFpEF patients.

## Materials and Methods

### Data

#### Framingham Heart Study

The FHS data used in this study included clinical, survival, and expression data downloaded from dbGAP (study accession: phs000007, http://dbgap.ncbi.nlm.nih.gov). The FHS has recruited participants from Framingham, MA, United States, to undergo biennial examinations to investigate cardiovascular disease and its risk factors since 1948 (Oppenheimer, 2005). Offspring (and their spouses) and adult grandchildren of the original cohort of participants were recruited into the second- and third-generation cohorts in 1971 and 2002, respectively (Yao et al., 2015). In this study, the clinical and gene expression data were obtained from the offspring cohort who (i) attended the eighth examination cycle conducted between 2005 and 2008 and (ii) had both clinical and gene expression profiles.

#### HFpEF Patients

According to the guidelines of the European Society of Cardiology (McMurray et al., 2018), patients were diagnosed with HFpEF using the following four conditions: (1) typical signs or symptoms of HF, (2) B-type natriuretic peptide >35 pg/ml and/or N-terminal-pro hormone B-type natriuretic peptide >125 pg/ml, (3) left ventricular ejection fraction >50%; and (4) structural HF (left ventricular hypertrophy/left atrial enlargement) and/or diastolic dysfunction. We excluded patients with valvular stroma and/or hypertrophic cardiomyopathy, resulting in inclusion of 172 HFpEF patients (103 males and 69 females). Patients whose 3-year survival status was unknown were filtered out by design (Fransen et al., 2011). Finally, 149 individuals (91 males and 58 females) who had full survival information after 3 years were included in the study.

#### Gene Expression Data

The expression data contained 17,873 gene expression probes. We mapped these probes to genes following the annotation from the Affymetrix Human Exon 1.0 ST GeneChip platform, which yielded 17,358 genes. The gene expression data were *log*_2_ (*x* + 1) transformed and then standardized (Cheerla and Gevaert, 2017). A variable screening procedure called as sure independence screening was applied to reduce the gene expression dimensionality from an ultra-high to a moderate scale, with a binary response defined as a “good outcome” or “poor outcome” for each individual. Following the sure independence screening criterion {i.e., keeping *d* = [2*n*/*log*(*n*)] features; Fan and Lv, 2008}, the top 137 features were retained for further analysis.

#### Clinical Outcome

The clinical outcome was defined as a good or poor outcome based on patients’ survival status. The good-outcome group had event-free survival for at least 3 years [survival time was measured from the time of admission for HFpEF diagnosis to the time of last follow-up (2011) or time of death from cardiovascular disease]. The poor-outcome group included patients who died because of cardiovascular disease during the 3-year period. We further explored the differentially expressed genes (DEGs) between the good-outcome and poor outcome groups using significance analysis of microarrays (Tusher et al., 2001) and then conducted Gene Ontology (GO) enrichment analysis and the Kyoto Encyclopedia of the Genes and Genomes (KEGG) pathway analysis based on the DEGs using KOBAS software^1^ (Ai and Kong, 2018).

### Statistical Analysis

#### KPLS Prediction Model Optimized With the Genetic Algorithm

The kernel partial least squares method can map the original data points from the original input space *R^N^* into a high-dimensional feature space *ℱ*, and therefore, original data that cannot be linearly separated in *R^N^* can be separated in *ℱ* (Rosipal and Trejo, 2002), which improves the classification performance to achieve accurate prediction. A genetic algorithm (GA) is an optimization method based on the genetic mechanism of “survival of the fittest.” In this study, we used a Gaussian kernel function to construct the kernel matrix for gene expression data and then used the genetic algorithm to optimize the Gaussian kernel function parameter *σ*. The Gaussian kernel function is given as $K\left(x_{i},x_{j}\right)=\exp\left(-{\left\|x_{i}-x_{j}\right\|}^{2}∕2\sigma^{2}\right)$. For the details of the method, readers are referred to Yang et al. (2020). Because we only used gene expression data for prediction, the only parameter that needed to be optimized was the kernel bandwidth σ.

#### Other Prediction Models

Ridge regression and LASSO fit prediction models by shrinkage or regularization of the regression coefficients (Frank and Friedman, 1993; Tibshirani, 1996). The LASSO method can shrink some coefficients to exactly zero. Both models were developed to minimize prediction errors. For the LASSO and RR methods, the optimal tuning parameter *λ* was chosen by 10-fold cross-validation over a grid of 100 λ values. The RR and LASSO methods were performed using the R glmnet package.

The SVM method was developed to solve high-dimensional classification problems (Furey et al., 2000) and was performed using the R e1071 package. The radial basis kernel function was used in the SVM.

An RF uses the bootstrap method to extract *n* samples from the original data and generate *B* classification trees. These *B* trees constitute a random forest. Each observation’s predictive result is determined by a majority vote; the overall prediction is the most commonly occurring class among the *B* classification trees (Austin et al., 2013). The RF method was performed using the randomForest package in R. All parameter values were set using the default.

#### Model Training and Testing

In our study, the original data were divided into two non-overlapping data sets: modeling data and external testing data. We randomly selected modeling data and external testing data at a ratio of 80:20. The modeling set was used to train the prediction model, and the testing set was used to evaluate the prediction performance. The entire process of randomly selecting the modeling and testing data was repeated 1,000 times to increase the stability and repeatability of the results.

#### Model Performance

We used multiple evaluation criteria to evaluate the predictive performances of the six models, including the area under the curve (AUC), sensitivity (Se), specificity (Sp), accuracy (ACC), Youden index, G-means, and Matthews correlation coefficient (MCC). The MCC and AUC were mainly used to evaluate the model performance because they are more comprehensive evaluation criteria. We employed one-way ANOVA, followed by Dunnett’s multiple-comparison test, to compare the performance of the GA-KPLS and the five other models (RF, LASSO, RR, Logit, and SVM). Statistical significance was indicated by a value of *p* < 0.05.

## Results

### Characteristics of HFpEF Patients in the FHS

At the end of the 3-year period, 42 patients (28.19%) met the study endpoint of cardiovascular disease-related death, and 107 patients (71.81%) had survived. There were 91 males (61.07%) and 58 females (38.93%). The average age was 75.02 (±8.02) years old. Table 1 shows the baseline condition of both groups, patients with good outcomes, and those with poor outcomes. There was no significant difference in age, gender, comorbidities, vital signs, or laboratory data (except for systolic blood pressure) between the two groups.

**Table 1:** Clinical characteristics of the study population (*N* = 149).

Characteristic	Good-outcome group (107)	Poor-outcome group (42)	*𝜒*^2^/*t*	p-value
Age, years	74.44 ± 8.23	76.50 ± 7.46	0.572	0.568
Female, *n* (%)	40(37.4)	18(42.9)	0.380	0.538
Comorbidities, *n* (%)				
Hypertension	84(78.5)	33(78.6)	<0.001	0.993
Hyperlipidemia	70(65.4)	26(61.9)	0.163	0.687
Diabetes	27(25.2)	11(26.2)	0.015	0.904
Vital signs and laboratory data				
Systolic blood pressure, mmHg^*^	127.74 ± 18.44	138.88 ± 22.71	−3.102	0.002
Diastolic blood pressure, mmHg	65.64 ± 11.58	67.83 ± 9.55	−1.08	0.279
Body mass index, kg/m^2^	29.84 ± 5.47	29.21 ± 5.68	0.633	0.528
Serum creatinine, mg/dl	1.24 ± 0.86	1.29 ± 0.88	0.288	0.774
Total cholesterol, mg/dl	162.12 ± 36.70	167.74 ± 41.31	−0.811	0.419
Heart rate, bpm	62.50 ± 10.90	64.45 ± 12.97	−0.929	0.354

* Shows the statistical significance at the α = 0.05 level.

### Model Performance Comparison

**Figure 1:**
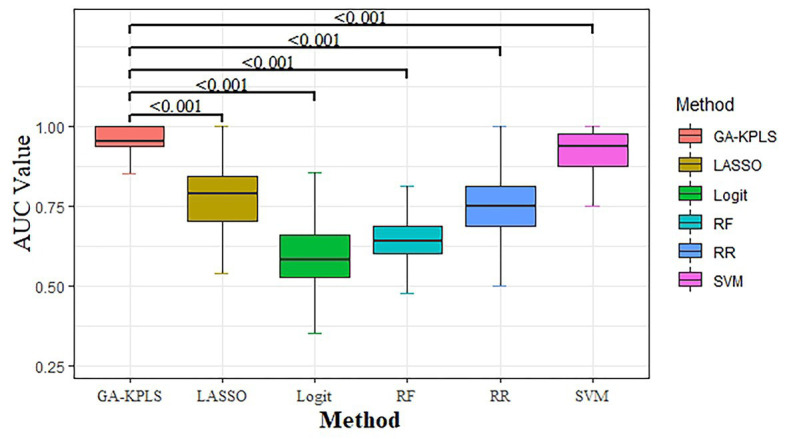
Boxplot of the area under the curve (AUC) values for the six different models (based on 1,000 random splits). The *y*-axis represents the AUC value. Values of *p* were obtained using Dunnett’s multiple-comparison test.

We compared the classification performance of the six models: GA-KPLS, RF, LASSO, RR, SVM, and Logit. The evaluation index of the six models was summarized as the average value obtained by repeating the data partition 1,000 times. Table 2 shows the prediction results of the six models. As shown in the table, the GA-KPLS model exhibited the best performance in nearly all the criteria except for specificity. This finding clearly demonstrates the superior performance of the GA-KPLS model. To further display the prediction results, we chose the evaluation criterion AUC to demonstrate the performance obtained by 1,000 random splits (see Figure 1). The AUC of the GA-KPLS model was significantly different from those of the RF, LASSO, RR, Logit, and SVM models, indicating the superior performance of the GA-KPLS model over the other models. It is interesting to note that the performance of the SVM model was quite similar to that of the GA-KPLS model. Based on the results, we concluded that the risk prediction model constructed by the GA-KPLS method had the best performance and can provide a methodological reference to assess the risk of HFpEF.

**Table 2:** Model performance.

Model	Se	Sp	AUC	ACC	Youden	F-measure	MCC	G-means
GA-KPLS	0.925	0.984	0.955	0.968	0.909	0.939	0.921	0.953
RF	0.319	0.974	0.646	0.793	0.293	0.445	0.427	0.535
LASSO	0.605	0.943	0.774	0.850	0.548	0.678	0.608	0.745
RR	0.469	1.000	0.734	0.853	0.469	0.618	0.620	0.669
Logit	0.549	0.574	0.591	0.567	0.122	0.410	0.112	0.548
SVM	0.870	0.989	0.929	0.956	0.859	0.913	0.891	0.926

### Prediction Result of HFpEF Using the GA-KPLS Method

**Figure 2:**
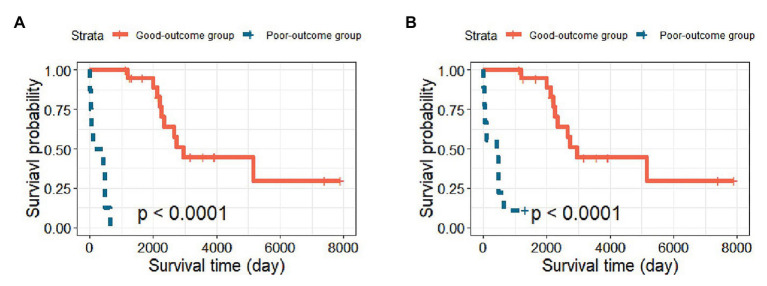
Kaplan-Meier survival curves of the good-outcome and poor-outcome groups. **(A)** The survival curve including the original 29 patients in the testing cohort and **(B)** the survival curve based on the predicted survival outcomes using the GA-KPLS method.

To demonstrate the clinical significance of identifying high-risk patients, we selected the prediction result of one random split with 120 training samples and 29 testing samples, which gave an MCC = 0.920 (close to MCC_mean_ = 0.921). The Kaplan-Meier curves based on the original and predicted data yielded significantly different survival probabilities (*p* < 0.0001). Figure 2 shows the survival curves of the two groups. The left panel shows the survival curve from the original data, and the right panel shows the survival curve based on the newly predicted risk group with the GA-KPLS method. The prediction method exhibited good performance because the survival curves using the original and predicted values were very similar. To predict a future event, all the data can be used as the training set, and then the risk group status can be predicted based on measured gene expression data.

### DEGs Between the Good-Outcome and Poor-Outcome Patients

**Figure 3:**
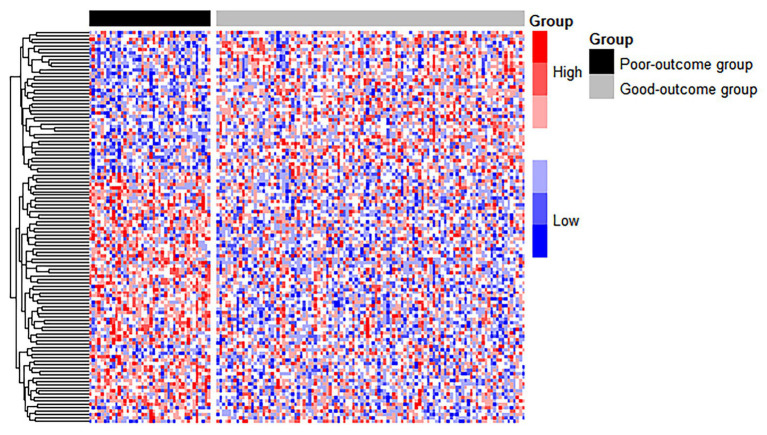
The heatmap of DEGs between the good-outcome and poor-outcome groups. Each column represents a patient, and each row represents a gene. Patients labeled with the black bar are poor-outcome samples, and those with the gray bar are good-outcome samples.

We treated the good-outcome group as the control group to identify DEGs. Of a total of 137 top genes, 116 DEGs were identified based on a threshold value of *q* < 0.05, among which 70 genes were upregulated and 46 were downregulated. The significant features of gene expression are shown in a heat map (see Figure 3). A block-like structure can be observed between the good-outcome and poor-outcome groups.

Among the 116 DEGs, the *TRAF3IP2*, *C1QTNF9*, *TECRL*, and *Eph* genes have been reported to be associated with HF. *TRAF3IP2* is an upstream regulator of multiple proinflammatory pathways. *TRAF3IP2* overexpression may activate IKK/NF-B, p38 MAPK, and JNK/AP-1 and induce proinflammatory cytokines, leading to cardiac fibrosis and contractile dysfunction (Yariswamy et al., 2016). *C1QTNF9* (*CTRP9*) is an important member of the *CTRP* protein family. Appari et al. (2016) found that *C1QTNF9* knock-out mice were protected from left ventricular dilatation and contractile dysfunction; however, *C1QTNF9* overexpression promoted ventricular remodeling and systolic dysfunction. *TECRL* was recently suggested to play a key role in the electrical activity of the heart. *TECRL* affects the electrical conduction system of the heart by causing mutations in a calcium-processing protein, which eventually leads to arrhythmia (Perry and Vandenberg, 2016). The Eph/ephrin receptor ligand comprises the largest family of receptor tyrosine kinases and affects the behavior of cells mainly by activating signal transduction pathways. Eph/ephrin expression may lead to phenotypic changes in the vascular endothelium during inflammation, causing inflammatory cells to enter the interstitial tissue from the vascular space (Coulthard et al., 2012).

The role of *DUSP1* is controversial, as both anti-inflammatory and pro-atherosclerotic actions have been suggested (Hahn et al., 2014). Auger-Messier et al. (2013) suggested that the disruption of *DUSP1* promoted p38 MAPK activity, which could reduce cardiac contractility and calcium handling; thus, *DUSP1* could be a target gene for prevention of HF. In addition, *LHFPL2* and *SNX24* are associated with coronary artery disease (Lin et al., 2013; Shendre et al., 2017). *HIST1H4B* is associated with the immune process (Zhang et al., 2019). *OXER1* is involved in the inflammatory response of the disease (Dattilo et al., 2015). The empirical evidence suggests the importance of the identified DEGs associated with HFpEF.

### Functional Analysis of DEGs

**Figure 4:**
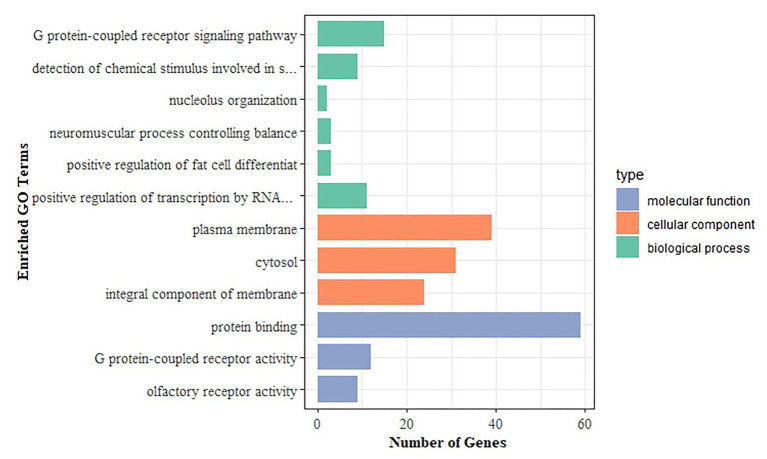
Gene Ontology (GO) enrichment analysis of DEGs. The x-axis shows the number of genes, and the y-axis indicates the GO terms. Bars with different colors correspond to different GO categories, with green representing biological process, orange representing cellular component, and blue representing molecular function.

To further investigate the functional relevance of the DEGs, we performed GO enrichment and KEGG pathway analyses. The DEGs were significantly enriched in 12 GO terms, with a *corrected value of p* < 0.05. GO terms comprised three categories: biological process, cell component, and molecular function. Figure 4 shows all significant GO terms. The most significantly enriched GO terms were plasma membrane (*corrected value of p* = 2.67E−07), G protein-coupled receptor signaling pathway (*corrected value of p* = 3.06E−04), and protein binding (*corrected value of p* = 3.06E−04). The plasma membrane plays important roles in maintaining homeostasis, cell material exchange, and information transmission (Lutz et al., 2003; Wang et al., 2017). The G protein-coupled receptor signaling pathway mediates cardiac functions, such as those of inotropy and vasodilation in peripheral vessels, participates in the occurrence and development of HF and may serve as the molecular underpinning for future HF therapeutics (Wang et al., 2018; Altamish et al., 2020). Protein binding, including fatty acid-binding proteins, has been related to cardiac alterations, e.g., systolic and diastolic cardiac dysfunction (Rodriguez-Calvo et al., 2017). In the KEGG analysis, the olfactory transduction pathway was identified, with a *corrected value of p* < 0.05. The olfactory system uses G protein-coupled receptors to accomplish its vital task (Ronnett and Moon, 2002).

## Discussion

Accurately predicting disease outcomes are essential for patient-centered care, both for making treatment decisions and monitoring the quality of health care (Angraal et al., 2020). Using the gene expression data of HFpEF patients, this study explored five machine learning methods and one conventional logistic regression model to predict the survival status of patients with HFpEF. The GA-KPLS based HFpEF model could predict patient survival status with high accuracy. Furthermore, the identification of molecular markers (i.e., DEGs) of HFpEF may lead to the development of novel targeted therapies.

The ability to assess survival outcomes of patients with cardiovascular diseases has great clinical value in an era with multiple treatment options. Although previous studies have devoted great effort to predicting clinical outcomes of HF patients, the current study has several unique merits. There are many studies being conducted to predict HF. However, few studies are focused on HFpEF. By evaluating six models, we showed that the GA-KPLS model using gene expression data may be a powerful and highly accurate prediction model of survival status in HFpEF patients. A prediction model using gene expression data can be an alternative means to the currently used models based on clinical data, such as the Enhanced Feedback for Effective Cardiac (EFFECT) study risk scores (Thorvaldsen et al., 2017) and Meta-Analysis Global Group in Chronic Heart Failure (MAGGIC) scores (Pocock et al., 2013).

Second, because of the highly heterogenous nature of HFpEF, a consensus has not been reached on which predictors can be used to reliably predict HFpEF. We demonstrated that gene expression can be used to predict HFpEF survival status with high accuracy using the GA-KPLS prediction model. With the availability of increasing types of omics data (e.g., copy number variants, microRNAs, and epigenetic data), we can further improve the prediction accuracy by integrating different data sources with the GA-KPLS model. Our study illustrates the development of new machine learning methods for HFpEF risk prediction by integrating different omics data types.

Current studies have focused on single or multiple clinical indicators to identify patients at high risk for HFpEF. However, most methods can only achieve an AUC of 0.7, which is unrealistic for application in clinical practice (Kanda et al., 2018; Shen et al., 2020). Many researchers have also used statistical methods to construct stratification models such as Cox proportional hazards models and logistic regression models. However, these methods fail to capture the nonlinear relationship between predictors and the disease outcome (Komajda et al., 2011; Rich et al., 2018; Angraal et al., 2020). In contrast, the GA-KPLS model uses the advantage of kernel functions to extract nonlinear relationships between genomic features and survival outcomes, hence achieving more accurate predictions than its counterparts.

Risk prediction in HFpEF patients using the GA-KPLS model may (1) serve to motivate patients to adhere to recommended treatments and lifestyle modifications (Oktay et al., 2013); (2) help clinicians to make treatment decisions, especially for high-risk groups of patients who may progress to circulatory failure when administered routine clinical therapeutics, and these patients may have the opportunity to undergo active therapeutic interventions such as mechanical circulatory assistance, heart transplantation, or new trials (Wang et al., 2019); and (3) help to inform the design of future HFpEF clinical trials.

However, our study had some limitations. First, because of the lack of additional external data on HFpEF, we cannot validate our findings in another data set. Second, we focused on gene expression data in our study. As lifestyle is an important risk factor for HF, further research should be performed to predict HFpEF risk by integrating both clinical and genomic data to improve the prediction performance because potential interactions may exist between these factors. Third, the HFpEF data set is imbalanced, with a ratio of 28:72 between the poor-outcome and good-outcome groups. However, the GA-KPLS and SVM methods performed well, with high sensitivity and specificity. If either low sensitivity or specificity becomes a concern, the SMOTE algorithm can be applied (Chawla et al., 2002), which is designed to handle prediction with imbalanced data.

In conclusion, the GA-KPLS-based HFpEF prediction model using gene expression data represents a valuable tool to improve the prognosis of HFpEF patients with different risk levels. The discovered transcriptional biomarkers of HFpEF provide new insight to the understanding the complex mechanism of HFpEF, leading to the development of novel targeted therapies for HFpEF. It is expected that integrating multi-omics and clinical data can further improve HFpEF outcome prediction, leading to the development of targeted, adaptive, and precision treatment of HFpEF patients with different risk levels.

## Data Availability Statement

The data analyzed in this study is subject to the following licenses/restrictions: The data analyzed in this study require NIH approval through the dbGap website. Requests to access these datasets should be directed to http://dbgap.ncbi.nlm.nih.gov.

## Author Contributions

LZ and ZG performed the study and drafted the manuscript. BW, YW, ZL, RF, and HtY participated in the data processing and analysis. HY provided the clinical interpretation. HC and YC conceived of the idea and revised the manuscript. All authors contributed to the article and approved the submitted version.

### Conflict of Interest

The authors declare that the research was conducted in the absence of any commercial or financial relationships that could be construed as a potential conflict of interest.

## Footnotes

^1^http://kobas.cbi.pku.edu.cn
